# *Foxc2* is essential for podocyte function

**DOI:** 10.14814/phy2.14083

**Published:** 2019-05-06

**Authors:** Daniel Nilsson, Mikael Heglind, Zahra Arani, Sven Enerbäck

**Affiliations:** ^1^ Department of Medical Biochemistry and Cell Biology Institute of Biomedicine Sahlgrenska Academy University of Gothenburg Gothenburg Sweden

**Keywords:** *Foxc2*, *Nrp1*, podocyte, proteinuria

## Abstract

*Foxc2* is one of the earliest podocyte markers during glomerular development. To circumvent embryonic lethal effects of global deletion of *Foxc2*, and to specifically investigate the role of *Foxc2* in podocytes, we generated mice with a podocyte‐specific *Foxc2* deletion. Mice carrying the homozygous deletion developed early proteinuria which progressed rapidly into end stage kidney failure and death around postnatal day 10. Conditional loss of *Foxc2* in podocytes caused typical characteristics of podocyte injury, such as podocyte foot process effacement and podocyte microvillus transformation, probably caused by disruption of the slit diaphragm. These effects were accompanied by a redistribution of several proteins known to be necessary for correct podocyte structure. One target gene that showed reduced glomerular expression was *Nrp1*, the gene encoding neuropilin 1, a protein that has been linked to diabetic nephropathy and proteinuria. We could show that NRP1 was regulated by *Foxc2* in vitro, but podocyte‐specific ablation of *Nrp1* in mice did not generate any phenotype in terms of proteinuria, suggesting that the gene might have more important roles in endothelial cells than in podocytes. Taken together, this study highlights a critical role for *Foxc2* as an important gene for podocyte function.

## Introduction

The forkhead gene *Foxc2* is a transcription factor with many vital functions during development, including skeletogenesis and patterning of the aortic branch (Iida et al. 1997). *Foxc2* expression has also been identified in podocytes (Dagenais et al. 2004; Takemoto et al. 2006; Brunskill et al. 2011) and it appears as one of the earliest podocyte markers required for correct glomerular development (Takemoto et al. 2006). Using a global knockout mouse model, it has been shown that loss of *Foxc2* results in downregulation of slit diaphragm‐associated NPHS2 (podocin) as well as collagen IV subunits a3 and a4 (COL4A3 and COL4A4) (Morello et al. 2001; Takemoto et al. 2006), two important components of the glomerular basement membrane (Miner and Sanes 1996; Korstanje et al. 2014). Additionally, the *Foxc2* global knockout had reduced glomerular levels of important factors such as rhophilin 1 (*Rhpn1*) and PODXL (Takemoto et al. 2006). In humans, mutations in *FOXC2* have been shown to cause lymphedema‐distichiasis syndrome, which in rare cases also result in renal disease (Fang et al. 2000; Erickson et al. 2001; Brice et al. 2002; Yildirim‐Toruner et al. 2004). Only heterozygosity of nonsense human *FOXC2* mutations have been reported pointing to a requirement of functional FOXC2 for proper development, a hypothesis strengthened by the lethal effects of complete *Foxc2* deletion in mice (Iida et al. 1997; Winnier et al. 1997).

Although global knockout mouse models are useful in studying gene functions, developmental or systemic effects in these models could mask a potential later role in differentiated cells and cell‐specific requirements cannot be addressed. The latter problem is highlighted in the case of *Lmx1b*, another transcription factor found in podocytes (Chen et al. 1998; Dreyer et al. 1998). Similar to *Foxc2*, global knockout of *Lmx1b* in mice causes glomerular downregulation of NPHS2, COL4A3, and COL4A4 (Miner et al. 2002). However, these effects on gene expression could not be confirmed when *Lmx1b* was specifically deleted in podocytes even though the kidney phenotype was profound (Suleiman et al. 2007; Burghardt et al. 2013). This emphasizes the caution one should take when interpreting data from global knockout mouse models.

Conditional deletion of genes, using the Cre‐lox system (Hoess et al. 1984), has proven to be an efficient way to circumvent postnatal lethality and developmental issues. Efforts have previously been made to conditionally delete *Foxc2* in the kidney, using either *Pax2* or *Nephrin* promoter to drive *Cre* expression (Motojima et al. 2016a, 2017). However, PAX2 is predominantly expressed in undifferentiated podocytes (Bariety et al. 2006), leaving the specific role of *Foxc2* in differentiated podocytes in vivo unknown, whereas *Nephrin* promoter has been found to be active not only in podocytes but also in brain and pancreas (Moeller et al. 2000; Putaala et al. 2001).

To be able to study the specific role of *Foxc2* in podocytes, we decided to generate mice with conditional *Foxc2* knockout using Podocin‐Cre transgenic mice (Moeller et al. 2003), a widely used model for podocyte‐specific genome modifications.

## Methods

### Mice

Generation of *Foxc2* ^lacZ/+^ mice was described earlier (Cederberg et al. 2009). Transgenic Pod‐Cre mice (aliases Nphs2‐Cre or 2.5P‐Cre) were a kind gift from L. Holzman (Moeller et al. 2003). Rosa26 Cre reporter strain (#3474) (Soriano 1999), and floxed *neuropilin 1* (*Nrp1*) (#5247) (Gu et al. 2003) mice were obtained from Jaxmice. These strains were maintained on a C57Bl/6 background.

Mice with a floxed *Foxc2* allele were generated by introducing loxP sites on each side of the single exon of *Foxc2*. Genomic DNA for the *Foxc2* locus was retrieved from a mouse 129/SvJ genomic library (Stratagene) and subcloned into the pPGKneobpAlox2PGKDTA vector. The 5′ loxP site was inserted into the BspEII‐site upstream of the start codon, where no known regulatory elements were identified. To avoid disruption of miRNA‐binding sites, the floxed Neo‐cassette was inserted outside of the 3′UTR. The targeting vector was linearized with NotI and electroporated into RW4 ES cells and homologous recombination was confirmed by southern blot. The Neo‐cassette was then removed by transient transfection of correctly targeted ES clones and positive clones were injected into the blastocyst obtained from C57Bl/6 mice. Chimeric mice were mated with C57Bl/6 mice to generate *Foxc2* ^fl/+^ mice.

To generate podocyte‐specific *Foxc2* or *Nrp1* knockouts, as well as Cre reporter mice, animals with floxed alleles were crossed with Pod‐Cre transgenic mice. The strain was maintained on a mixed sv129;C57Bl/6 background.

All animal procedures were approved by the Ethical Committee for Animal Experiments at the University of Gothenburg, which adheres to the principles and guidelines established by the European Convention for the Protection of Laboratory Animals.

### Genotyping

Alleles for *Foxc2* ^lacZ^, Pod‐Cre transgene, Rosa26^STOPlacZ^, and floxed *Nrp1* were genotyped as previously described (Soriano 1999; Gu et al. 2003; Moeller et al. 2003; Cederberg et al. 2009). Floxed *Foxc2* allele was genotyped by PCR using primers located on each side of the inserted 5′ loxP site (sense primer 5′‐ AAACTCGCTTTGAGCCAGAA ‐3′ and antisense primer 5′‐ CCCTTCCGAGTGCTAGAAAA‐3′). Expected products were 180 bp from the wild‐type allele and 220 bp from the floxed *Foxc2* allele. Genotyping of the null allele of *Nrp1* was performed by PCR, using sense primer 5′‐ AAGGAGTGGCACAGCATCTT‐3′ and antisense primer 5′‐ TTGGGTGAACTCAGCCACTT‐3′. Amplification from the *Nrp1* null allele was estimated to generate a 350 bp PCR product, whereas wild‐type allele should generate a 739 bp band.

### Histology and morphology

Embryos and kidneys for X‐gal staining and standard histology were harvested and immersed in ice‐cold 4% (w/v) paraformaldehyde (PFA)(Sigma‐Aldrich) in phosphate‐buffered saline (PBS)(Medicago) for 1 h. Fixed specimens for cryosectioning were immersed in ice‐cold 30% (w/v) sucrose in PBS until they sunk, embedded in OCT Cryomount (Histolab) and cryosectioned at 10 *μ*m thickness.

For detection of *β*‐galactosidase expression, fixed whole embryos or kidneys were incubated in 5‐bromo‐4‐chloro‐3‐indolyl‐*β*‐D‐galactopyranoside (X‐Gal) staining solution (5 mmol/L K_3_Fe(CN)_6_ (ICN), 5 mmol/L K_4_Fe(CN)_6_ (ICN), 5 mmol/L EGTA (Sigma‐Aldrich), 0.01% (w/v) deoxycholate (Sigma‐Aldrich), 0.02% (v/v) NP‐40 (BDH), 2 mmol/L MgCl2 (Scharlau), and 1 mg/mL of X‐Gal (Sigma‐Aldrich) in PBS) overnight at 37°C. Here, *β*‐galactosidase‐expressing tissues were located and photographed under a dissecting microscope.

Stained embryos were postfixed in 10% (v/v) formalin and embedded in a mixture of 25% (w/v) bovine serum albumin (BSA)(Sigma‐Aldrich) and 0.4% (w/v) gelatin in PBS. The solution was solidified by addition of glutaraldehyde (Sigma‐Aldrich) to a final concentration of 2.5% (v/v), and the resulting tissue blocks were serial sectioned on a vibratome at 30 *μ*m. Sections were mounted on SuperFrost microscope slides and counterstained for 5 min in 0.1% (w/v) cresyl violet acetate (Sigma‐Aldrich) in distilled H_2_O, and washed for 15 min in 96% ethanol (Solveco) and for 5 min in PBS.

Standard hematoxylin and eosin staining for histology was performed on cryosections using Mayer′s HTX (Histolab) and eosin Y with phloxine (Sigma‐Aldrich). Histochemically stained sections were mounted in Canada Balsam (Sigma‐Aldrich), and then examined and photographed using an Eclipse E800 microscope (Nikon).

Kidneys for electron microscopy were fixed overnight in modified Karnovsky′s fixative (2.5% (v/v) glutaraldehyde, 2% (w/v) paraformaldehyde, 0.05 mol/L sodium cacodylate (Sigma‐Aldrich), 0.02% (w/v) sodium azide (Sigma‐Aldrich), pH 7.2) For transmission electron microscopy, kidneys were rinsed in 0.15 mol/L sodium cacodylate and postfixed in 1% (w/v) osmium tetroxide (OsO_4_) (Sigma‐Aldrich), and 1% (w/v) potassium ferrocyanide (K_4_Fe(CN)_6_) for 2 h at 4°C. Contrast was enhanced by incubation in 0.5% (w/v) uranyl acetate for 1 h in darkness at room temperature. After dehydration, the filters with attached cells were embedded in Agar 100 resin (Agar Scientific LTD). Ultrathin transverse sections (60–70 nm) of the filters with attached cells were contrasted with uranyl acetate and lead citrate before examination using a Zeiss LEO 912 Omega transmission electron microscope (Zeiss). Digital image files were captured with a MegaView III camera (Soft Imaging Systems).

For scanning electron microscopy, 60 *μ*m vibratome‐sections of kidneys were treated by modified osmium‐thiocarbohydrazide‐osmium method, that is, 2 h incubation in 1% (w/v) OsO_4_ at 4°C, 10 min in 1% (w/v) thiocarbohydrazide (Sigma‐Aldrich) followed by 1 h incubation in 1% (w/v) OsO_4_ at 4°C. Sections were dehydrated in ethanol followed by immersion in hexamethyldisilazane (Sigma‐Aldrich) and evaporation in a fume hood. The dried specimens were mounted on stubs and coated with palladium and then examined in a Zeiss 982 Gemini field emission scanning electron microscope equipped with an in‐lens secondary electron detector (Zeiss).

### Immunohistochemistry

Kidneys were dissected and embedded in OCT Cryomount (Histolab). Cryosections at 10 *μ*m were fixed and permeabilized in ice‐cold acetone (Fisher Scientific) for 10 min or fixed in 4% (w/v) paraformaldehyde in PBS for 20 min at room temperature and permeabilized in 0.2% (v/v) Triton X‐100 in PBS for 15 min at room temperature. Sections were then blocked in 10% (v/v) fetal bovine serum in DMEM medium (Gibco) (blocking solution) for 1 h at room temperature and incubated with primary antibody diluted in blocking solution at 4°C overnight. After washing in PBS, the sections were incubated with secondary antibody and TO‐PRO^®^‐3 nuclear stain (Thermo Fisher Scientific 1:1000) diluted in blocking solution for 1 h at room temperature. Sections were washed again in PBS and slides were mounted in ProLong^®^ Gold Antifade Mountant (Thermo Fisher Scientific) or ProLong^®^ Diamond Antifade Mountant (Thermo Fisher Scientific) and photographed using an LSM 510 Meta confocal microscope (Zeiss). Antibodies used are listed in Table 1.

**Table 1:** Antibodies used for detection of proteins. (n/a = not applicable, Thermo FS = Thermo Fisher Scientific). Higher dilution used for immunoblotting, lower for immunofluorescence

Antibody	Protein symbol	Host	Catalogue #	Company	Dilution
Wilm′s tumor protein	Wt1	Rabbit	ab15249	Abcam	1:200
KI67	Mki67	Rabbit	ab15580	Abcam	1:200
Actin, *α*‐Smooth Muscle FITC	Acta2	Mouse	F3777	Sigma‐Aldrich	1:200
Cd31	Cd31	Rat	ab7388	Abcam	1:200
Nephrin	Nphs1	Rabbit	ab58968	Abcam	1:100
Podocin	Nphs2	Rabbit	P0372	Sigma‐Aldrich	1:400
ZO‐1/TJP1	Tjp1	Rabbit	40‐2200	Thermo FS	1:100
Podocalyxin	Podxl	Goat	AF1556	R&D Systems	1:400
Alpha‐actinin 4	Actn4	Rabbit	0042‐5	Immunoglobe	1:200
Nestin	Nes	Rabbit	ab24692	Abcam	1:200
CD2‐associated protein	Cd2ap	Rabbit	ab32741	Abcam	1:400
Integrin beta‐1	Itgb1	Rat	MAB1997	Millipore	1:200;1:1000
Integrin linked kinase	Ilk	Rabbit	ab52480	Abcam	1:200
CCN1	Cyr61	Rabbit	ab24448	Abcam	1:100
Collagen IV subunit a3	Col4a3	Rat	7076	Chondrex	1:100
Collagen IV subunit a4	Col4a4	Rat	7073	Chondrex	1:100
Neuropilin 1	Nrp1	Goat	AF566	R&D Systems	1:200; 1:1000
Rabbit IgG Alexa 488	n/a	Donkey	A21206	Thermo FS	1:500
Rabbit IgG Alexa 568	n/a	Donkey	A10042	Thermo FS	1:500
Goat IgG Alexa 488	n/a	Donkey	A11055	Thermo FS	1:500
Rat IgG Alexa 488	n/a	Donkey	A‐21208	Thermo FS	1:500
Goat IgG HRP	n/a	Rabbit	P0449	Dako	1:2000
Rat IgG HRP	n/a	Goat	NA935	GE Lifescience	1:20,000

TUNEL staining was performed using Click‐iT Plus TUNEL assay with Alexa488 (Thermo Fisher Scientific, C10617) according to manufacturer's protocol.

### Image analysis

WT1 positive cells (according to labeling with WT1 primary antibody) were identified in glomeruli from either *Foxc2* ^fl/fl^ or *Foxc2* ^fl/fl^; Pod‐Cre^+/−^ mice and automatically counted using ImageJ software and the built‐in function of “Analyze particles”.

### Urine analysis

Spot urine was collected and 2 *μ*L of each urine sample, and BSA standards at 0, 1, 3, and 10 *μ*g, were separated by SDS‐PAGE on a NuPAGE Novex 4–12% Bis‐Tris gel (Thermo Fisher Scientific). After electrophoresis, the gel was immersed in fixing solution (50% (v/v) methanol (Fisher Scientific), 10% (v/v) acetic acid (Acros)) for 1 h, incubated for 20 min with agitation in staining solution (0.1% (w/v) Comassie brilliant blue R‐250 (Bio‐Rad), 50% (v/v) methanol, 10% (v/v) acetic acid), and finally incubated several times in destaining solution (40% (v/v) methanol, 10% (v/v) acetic acid) until destained.

Urinary albumin content in spot urine was assessed using Albuwell M indirect ELISA (Exocell) and urinary creatinine content was measured, using Creatinine Companion kit (Exocell). Both measurements were performed according to manufacturer′s instructions. Albumin:creatinine ratio was calculated by dividing *μ*g albumin/mL urine with mg creatinine/mL urine, getting the ratio *μ*g albumin/mg creatinine.

### Cell culture

An immortalized podocyte cell line E11 (Cell Lines Service GmbH) (Schiwek et al. 2004) was maintained at 33°C RPMI 1640 Medium, GlutaMAX™ Supplement (Gibco) supplemented with 100 U/mL penicillin‐streptomycin (Thermo Fisher Scientific) and 10 U/mL IFN*γ* (Millipore). For differentiation, IFN*γ* was excluded from the medium and cells were incubated at 37°C until harvesting.

For knockdown experiments, cells cultured at 33°C were transfected with either *Foxc2* ON‐TARGETplus siRNA pool (GE Dharmacon), or non‐targeting ON‐TARGETplus siRNA control (GE Dharmacon), using Lipofectamine RNAiMAX (Thermo Fisher Scientific) according to the manufacturer′s protocol. After transfection, cells were transferred to 37°C, in medium without IFN*γ*, to induce differentiation until harvesting after 48 h.

Overexpression of mouse *Foxc2* was achieved by retroviral transduction of undifferentiated E11 cells. Full‐length coding sequence of mouse *Foxc2* (NM_013519.2) were PCR‐amplified from genomic DNA (C57Bl/6) (primers used: sense 5′‐ AAAGGATCCTCTGGGACGCAGCATGCAGG‐3′ and antisense 5′‐ AAAGATATCTCAGTATTTGGTGCAGTCGTAAGA‐3′) and the PCR product was cloned in retroviral pBabe‐puro vector (gift from Hartmut Land & Jay Morgenstern & Bob Weinberg (Addgene plasmid # 1764)) (Morgenstern and Land 1990). Replication‐incompetent retroviruses were produced in 293T cells by co‐transfection of retroviral vectors together with pVPack‐GP and pVPack‐Eco packaging vectors (Agilent) and E11 cells was transduced essentially as described (Gerin et al. 2009).

### Isolation of glomeruli

Glomeruli were isolated using the magnetic bead isolation procedure essentially as described (Takemoto et al. 2002). Briefly, mice were anesthetized followed by perfusion (through the heart) with 2 × 10^7^ Dynabeads (Thermo Fisher Scientific) diluted in 10 mL of PBS. The kidneys were removed, minced, and digested in collagenase (1 mg/mL collagenase A (Roche), 100 U/mL deoxyribonuclease I (QIAGEN) in PBS) at 37°C for 15 min with gentle agitation. The collagenase‐digested tissue was gently pressed through a 100‐*μ*m cell strainer, washed with PBS and filtered through a new cell strainer without pressing, and the cell strainer was washed with PBS. The cell suspension was then centrifuged at 200*g* for 5 min and the cell pellet was resuspended in 2 mL of PBS. Finally, glomeruli containing Dynabeads were gathered by a magnetic particle concentrator and washed for at least three times with PBS.

### Gene expression analysis

Total RNA from glomeruli and E11 cells was isolated using RNeasy mini or plus micro kits (QIAGEN) according to the manufacturer's instructions. Reverse transcription of 1 *μ*g of total RNA was carried out using Transcriptor First‐Strand cDNA synthesis kit (Roche Life Science), according to the manufacturer′s instructions using random primers. Expression levels of specific mRNAs were quantified by real‐time PCR, using Power SYBR green PCR Master Mix (Thermo Fisher Scientific) on a ViiA™ 7 Real Time Detection System (Thermo Fisher Scientific) and normalized to the level of *Rplp0*. All samples were analyzed in quadruplicate, and mean values were calculated. Primers were designed using Primer‐BLAST software (Ye et al. 2012). The sequence of specific primers can be found in Table 2.

**Table 2:** Sequences for primers used in quantitative PCR analysis of gene expression

PrimerID	Sequence 5′ ‐ 3′
Foxc2 F	AACCCAACAGCAAACTTTCC
Foxc2 R	TATTTGGTGCAGTCGTAAGAG
Nphs1 F	ACCAACATCCAGCTCGTCAG
Nphs1 R	CCAGGTTTCCACTCCAGTCC
Nphs2 F	CAAGCCCTCTGGATTAGGGG
Nphs2 R	CCAGGACCTTTGGCTCTTCC
Rhpn1 F	TGATCCTTGAGGAGAGGCCA
Rhpn1 R	TTGCTGATCTGCTGGTGGAG
Synpo F	GTAGCCAGGTGAGCCAAGG
Synpo R	TGAACTCGTTCACCCTCTGC
Wt1 F	CACGGCACAGGGTATGAGAG
Wt1 R	GTTGGGGCCACTCCAGATAC
Actn4 F	CACAGGCCTGAGCTGATTGA
Actn4 R	CACGATGTCCTCAGCATCCA
Ilk F	GTGGCTGGACAACACAGAGA
Ilk R	CATTTCAACCACCGCAGAGC
Emx2 F	ACCGATATCTGGGTCATCGC
Emx2 R	GTTCGAATCCGCTTTGGCTT
Gdnf F	TGGGTCTCCTGGATGGGATT
Gdnf R	GCGCTTCGAGAAGCCTCTTA
Nrp1 F	AGCTTCGGACGTTTTCACCT
Nrp1 R	GGAAGTCATCACCTGTGCCA
Ppard F	GGGAAAAGTTTTGGCAGGAGC
Ppard R	CCTCTGAGTGAGTCCCATGC

### Western blot analysis

Cells were lysed in RIPA buffer (50 mmol/L Tris‐HCl (MP Biochemicals), pH 8.0, 1 mmol/L EDTA (Sigma‐Aldrich), 1% (v/v) Triton X‐100 (Sigma‐Aldrich), 0.5% (w/v) sodium deoxycholate (Sigma‐Aldrich), 0.1% (w/v) SDS (Serva), 150 mmol/L NaCl (Scharlau)), supplemented with Complete protease inhibitor cocktail (Roche Life Science) according to the manufacturer's recommendations. Protein concentrations were measured using the Pierce BCA protein assay kit (Thermo Fisher Scientific) and 20 *μ*g of total proteins were separated by SDS‐PAGE on NuPAGE 4–12% Bis–Tris protein gels (Thermo Fisher Scientific) and transferred to Immobilon‐PSQ membrane (Millipore). The membrane was blocked by incubation in blocking solution (5% (w/v) skim milk powder (Millipore) in tris‐buffered saline (TBS)). Specific proteins were detected with indicated primary antibodies (see Table 1). Horseradish peroxidase‐coupled secondary antibody was visualized with West Dura Chemiluminescent Substrates (Thermo Fisher Scientific) on a LAS‐4000 Luminescent Image analyzer (FujiFilm).

## Results

### *Foxc2* is widely expressed

**Figure 1:**
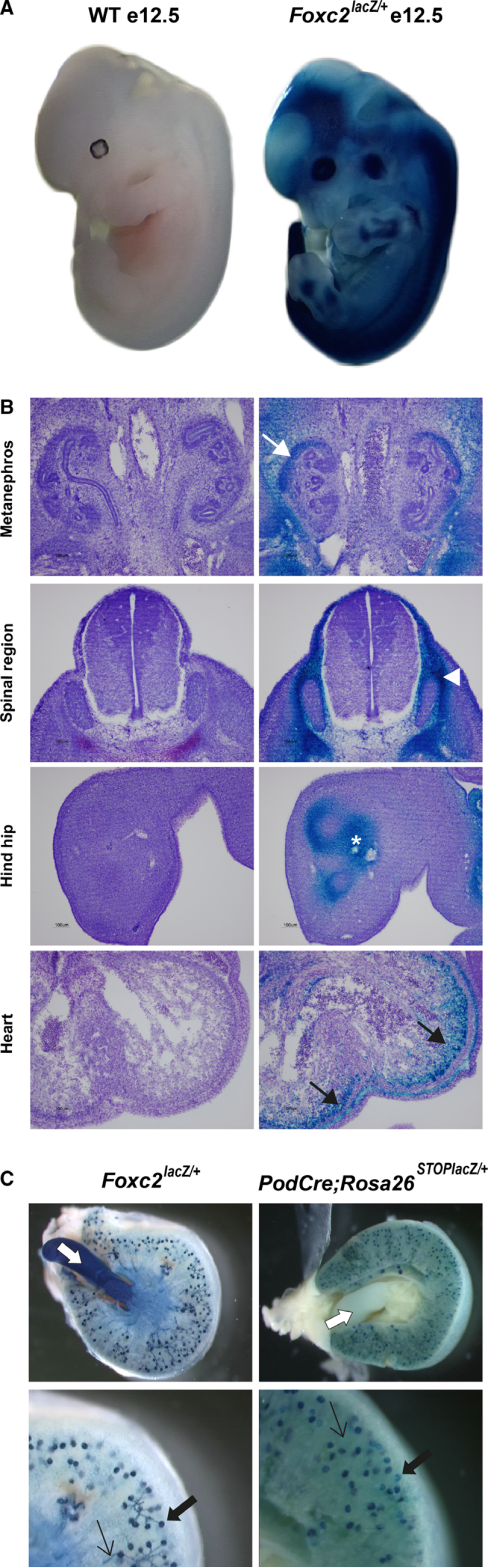
Characterization of lacZ expression. (A) Whole‐mount X‐gal staining Foxc2lacZ/+ and wild‐type (WT) embryos (day 12 postconception = e12.5). (B) Subsequent sectioning reveal blue X‐gal precipitate, as an indicator of active *β*‐galactosidase, in developing structures of mesenchymal origin, such as metanephros (white arrow), marginal layer of the spine (arrow head), precartilage primordium (asterisk) and myocardium (black arrows). No background staining was detected in control embryos. (C) *Foxc2* and *Cre* expression visualized by X‐gal staining in the adult kidney of two different *lacZ* mouse models. Cross‐sections of X‐gal‐stained kidneys, with close‐up. In adult *Foxc2* ^lacZ/+^ kidney, glomeruli/podocytes (black thick arrow), small vessels (black thin arrow) as well as ureter (white arrow) stain blue whereas in adult Pod‐Cre; Rosa26^STOP^ ^lacZ/+^ kidney, blue stain is visible in glomeruli/podocytes (black thick arrow) but not in vessels (black thin arrow) or ureter (white arrow).

Previous studies of the function of FOXC2 in mice are mainly based on global knockouts (Iida et al. 1997; Winnier et al. 1997; Takemoto et al. 2006), and due to embryonic lethality in these models, information on FOXC2 requirement beyond embryonic development is lacking. To identify cells expressing *Foxc2*, we have previously generated a knocked‐in mouse model where the single exon of *Foxc2* is replaced by the *lacZ* gene, encoding *β*‐galactosidase, resulting in *lacZ* expression under control of the endogenous *Foxc2* promoter (Cederberg et al. 2009). By performing X‐gal staining on *Foxc2* ^lacZ/+^ embryos at day 12 after conception (Fig. 1A), we could confirm previous reports showing that *Foxc2* is widely expressed during embryonic development (Miura et al. 1993; Kaestner et al. 1996). At embryonic day 12.5, *β*‐galactosidase activity was detected in the developing bone and vertebrae as well as in nephrons and the heart, indicating that the *Foxc2* promoter is active in these tissues (Fig. 1B).

### Podocyte‐specific deletion of *Foxc2* results in postnatal lethality and proteinuria

Further investigation of the kidney showed that, based on the X‐gal staining in adult *Foxc2* ^lacZ/+^ mice, *Foxc2* is expressed in several structures, including glomeruli, vessels, and ureter (Fig. 1C). This expression pattern makes cell‐specific roles of *Foxc2* in the kidney difficult to dissect, using the global knockout model. We were interested in studying the requirement of *Foxc2* specifically in the podocytes, so we sought to develop a different model. A well‐characterized model for podocyte‐specific deletions is the Pod‐Cre transgenic mouse (Moeller et al. 2003). By crossing this mouse with a *Rosa26* Cre‐reporter strain (Soriano 1999) and performing X‐gal staining, we could confirm that Cre activity was specific for glomeruli, whereas other structures are unstained (Fig. 1C).

To be able to study the role of *Foxc2* specifically in podocytes, we generated a mouse with loxP sequences flanking the single exon of *Foxc2* and crossed it with Pod‐Cre^+/−^ mice to achieve podocyte‐specific deletion of *Foxc2*. The *Foxc2* ^fl/+^; Pod‐Cre^+/−^ and *Foxc2* ^fl/fl^ control mice were viable, fertile and showed no obvious phenotype, indicating that neither the Cre‐transgene nor the presence of loxP sequences in potential regulatory regions of *Foxc2* were harmful to the mice.

**Figure 2:**
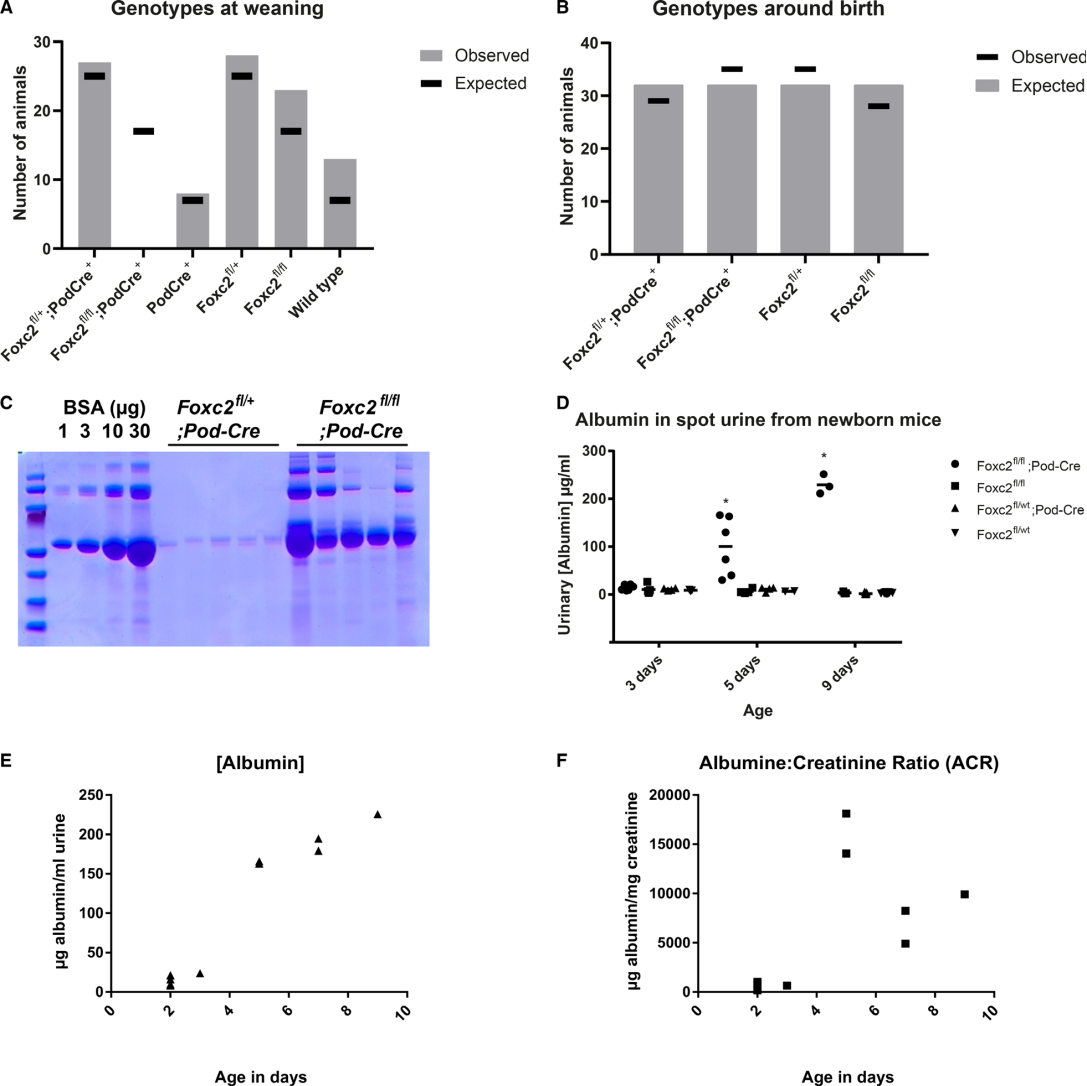
Characterization of podocyte‐specific *Foxc2* knockout mice. (A) Number of animals, observed and expected, with stated genotype out of 99 weaned pups from breedings set up to generate *Foxc2* ^fl/fl^; Pod‐Cre^+^ animals. Mating schemes for crossbreeding were either *Foxc2* ^fl/+^; Pod‐Cre^+^ × *Foxc2* ^fl/+^ (expected outcome: 12.5% *Foxc2* ^fl/fl^; Pod‐Cre^+^ animals) or *Foxc2* ^fl/+^; Pod‐Cre^+^ × *Foxc2* ^fl/fl^ (expected outcome: 25% *Foxc2* ^fl/fl^; Pod‐Cre^+)^. (B) Genotyping results for 127 pups up to 10 days of age, resulting from *Foxc2* ^fl/+^; Pod‐Cre^+^ × *Foxc2* ^fl/fl^ crossbreeding (expected outcome: 25% *Foxc2* ^fl/fl^; Pod‐Cre^+)^. (C) Analysis of urine samples (2 *μ*L) from 10 individual 5‐day‐old pups, genotyped as either *Foxc2* ^fl/+^; Pod‐Cre^+^ or *Foxc2* ^fl/fl^; Pod‐Cre^+^, on a Coomassie‐stained SDS‐PAGE gel to visualize protein content. BSA was included as standard at given amounts. (D) Measurement of albumin concentration in spot urine samples from newborn mice using enzyme‐linked immunosorbent assay (ELISA). (E) Measurement of albumin concentration (*μ*g albumin/mL urine) in spot urine samples from newborn *Foxc2* ^fl/fl^; Pod‐Cre^+^ mice from 2‐days till 9‐days‐of‐age. (F) Albumin:creatinine ratio (*μ*g albumin/mgcreatinine) for urine samples displayed in Figure 2E.

Mating for homozygous *Foxc2* ^fl/fl^; Pod‐Cre^+/−^ mice resulted in litters that, at the time of weaning (3–4 weeks of age), completely lacked the *Foxc2* ^fl/fl^; Pod‐Cre^+/−^ genotype (Fig. 2A), indicating embryonic or early postnatal lethality. Examination of pups from the late embryonic stage revealed that the predicted genotypes were present at normal ratio (Fig. 2B) confirming that the genetic modification is not causing embryonic lethality. Instead, the homozygous deletion of *Foxc2* in podocytes causes complete penetrance of postnatal lethality, since animals with this genotype die around postnatal day 10.

To assess the kidney function, urine from newborn mice was analyzed by SDS‐PAGE followed by Coomassie‐staining and revealed extensive proteinuria already at 5 days of age in homozygous *Foxc2* ^fl/fl^; Pod‐Cre^+/−^ mice, but not in heterozygous *Foxc2* ^fl/+^; Pod‐Cre^+/−^ mice (Fig. 2C). A more precise measurement of urinary albumin, using ELISA showed that there was no albuminuria in urine from 3‐day‐old pups, regardless of genotype (Fig. 2D). At postnatal day 5 and 9 however, the albuminuria could be confirmed in *Foxc2* ^fl/fl^; Pod‐Cre^+/−^ mice, whereas albumin levels were normal in the other genotypes tested (Fig. 2D). To compensate for differences in glomerular filtration rate, urinary albumin content is often normalized toward urinary creatinine content, getting an albumin:creatinine ratio (ACR). However, concerns have been raised that ACR is not suitable in diseases involving severe kidney injury (Waikar et al. 2010). Urinary albumin content was assessed in *Foxc2* ^fl/fl^; Pod‐Cre^+/−^ 2–9‐day‐old pups (Fig. 2E) and in parallel normalized toward the urinary creatinine levels (Fig. 2F). Urinary albumin increased with increasing age (Fig. 2E), whereas ACR makes a sudden drop at 7 days of age after substantial increase at 5 days of age (Fig. 2F). This discrepancy makes ACR an unsuitable method of choice to assess kidney function in this animal model with severe kidney injury.

### Loss of *Foxc2* in podocytes causes foot process effacement and microvillus transformation

**Figure 3:**
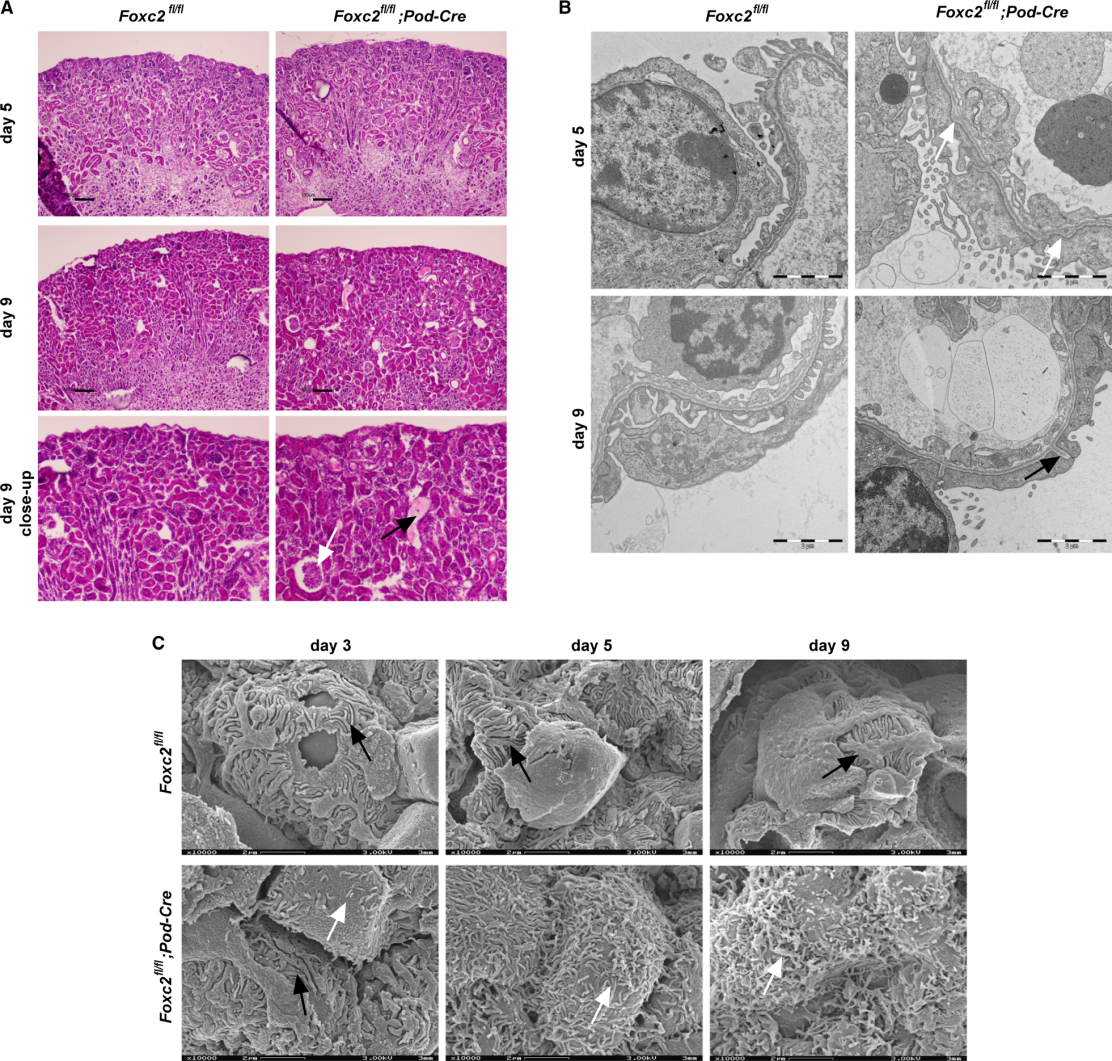
Morphology of kidneys from conditional *Foxc2* deletion in podocytes. (A) Histochemical (H&E) staining of kidneys from 5‐ and 9‐day‐old *Foxc2* ^fl/fl^ and *Foxc2* ^fl/fl^; Pod‐Cre^+^ mice with close‐ups of kidneys from 9‐day‐old mice. Enlarged glomerulus indicated with white arrow and tubular casts with black arrow. (B) Transmission electron micrographs (TEM) of kidneys from 5‐ and 9‐day‐old *Foxc2* ^fl/fl^ and *Foxc2* ^fl/fl^; Pod‐Cre^+^ mice. Splitting of basal membrane marked with white arrow and folding of basal membrane marked with black arrow. (C) Scanning electron micrographs of podocytes from *Foxc2* ^fl/fl^; Pod‐Cre^+^ and *Foxc2* ^fl/fl^ mice at postnatal day 3, 5 and 9. Podocyte foot processes marked with black arrow and microvilli‐like structures marked with white arrows. Scale bar (A) 100 *μ*m and (B–C) 2 *μ*m.

Kidneys in the global *Foxc2* knockout mice were markedly reduced (Takemoto et al. 2006), but this was not a characteristic shared by mice that had *Foxc2* specifically deleted in podocytes. Thus, most likely a phenotype not derived from podocytes. Comparison of kidney weight as % of body weight during the first ten postnatal days did not reveal any differences between the genotypes (Table 3). Although mice lacking *Foxc2* in the podocytes display extensive proteinuria already 5 days after birth, no morphological changes of the kidneys could be detected at this stage, using standard hematoxylin‐eosin histochemical staining. At 9 days of age, renal lesions, such as enlarged glomeruli and dilated tubuli with proteinaceous content became apparent (Fig. 3A).

**Table 3:** Kidney weight relative to body weight (b.w.) in newborn mice

Genotype	Kidney weight (% of b.w.)	StdDev
Foxc2^fl/+^;Pod‐Cre^+^	1.43	0.28
Foxc2^fl/fl^;Pod‐Cre^+^	1.37	0.1
Foxc2^fl/+^	1.30	0.12
Foxc2^fl/fl^	1.25	0.07

Ultrastructural investigation using transmission electron microscopy (TEM) revealed typical signs of podocyte injury. Mice lacking *Foxc2* expression in the podocytes exhibited foot process effacement (Fig. 3B). Occasional splitting of the basement membrane was seen at day 5, but could not be detected at later stages. However, at postnatal day 9, apparent folding of the basement membrane between effaced foot processes is observed (Fig. 3B). Other structural changes known to accompany podocyte injury, including thickening of basement membrane or loss of endothelial fenestrae; could not be detected, not even at postnatal day 9 when the mice suffer from end stage kidney failure (Fig. 3B).

TEM images also indicated the presence of microvillus‐like structures in the urinary space of the glomeruli of mice with podocyte‐specific *Foxc2* deletion. Scanning electron microscopy confirmed progression of podocyte microvillus transformation, that is, the presence of microvillus‐like structures extending from the cell body of podocytes that lack *Foxc2* expression (Fig. 3C). The loss of the typical podocyte structure of interdigitated foot processes was also evident.

### Mesangial expansion but no change in markers for apoptosis or proliferation after *Foxc2* deletion in podocytes

**Figure 4:**
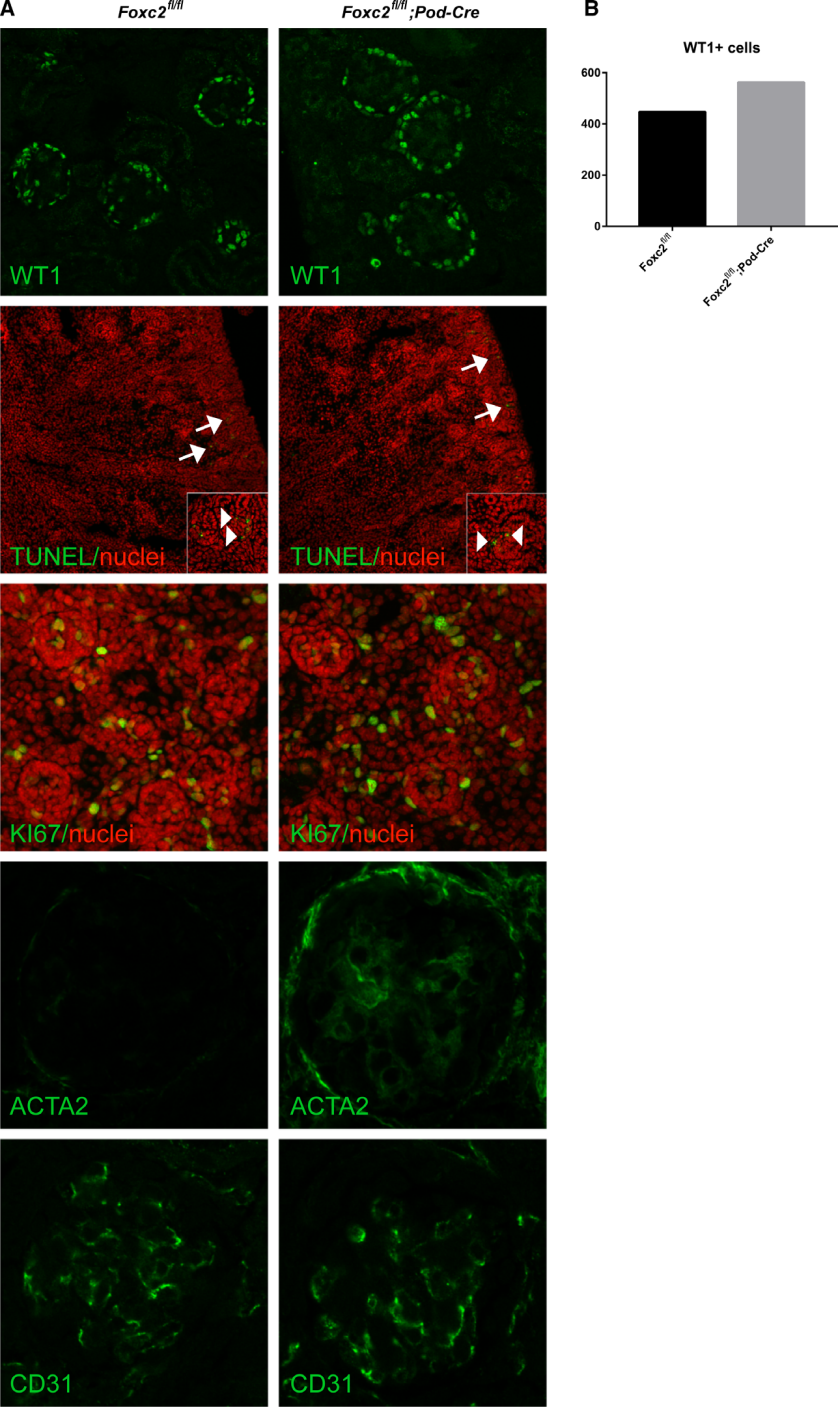
Effect of *Foxc2* deletion on podocyte number, proliferation, apoptosis and other glomerular cells. (A) Laser confocal microscopy of kidney sections from *Foxc2* ^*fl/fl*^ *;Pod‐Cre* ^+^ and *Foxc2* ^*fl/fl*^ mice labeled with primary antibodies against WT1 (podocyte marker), KI67 (dedifferentiation/proliferation marker), ACTA2/*α*‐SMA (mesangial cell marker), and CD31 (endothelial cell marker) (all green). TUNEL‐assay performed to identify apoptotic cells (green) in kidney (arrow) and glomeruli (arrow head in inset). TO‐PRO‐3 used for counterstaining of nuclei (red) in KI67 and TUNEL‐staining. (B) Counting of WT1 positive (WT1+) in cells in glomeruli (*n* = 43) from two different mice of each genotype.

Since *Foxc2* has been shown to have major roles in embryonic and podocyte development, knocking out *Foxc2* in the podocyte might affect several aspects of podocyte cell biology, such as proliferation, differentiation or even apoptosis; conditions that are known to cause foot process effacement, and proteinuria (Greka and Mundel 2012). Nevertheless, staining kidneys for markers for these features did not show any difference between *Foxc2* ^fl/fl^; Pod‐Cre^+/−^, and *Foxc2* ^fl/fl^ control mice (Fig. 4A). There was no indication of podocyte loss based on number of WT1 positive nuclei or degree of apoptosis detected by TUNEL‐assay in *Foxc2* ^fl/fl^; Pod‐Cre^+/−^ kidneys compared with *Foxc2* ^fl/fl^ control kidneys. A more thorough investigation of WT1 positive nuclei confirms the notion that there was no reduction in the number of podocytes (Fig. 4B). By labeling for KI67, a marker for proliferation/dedifferentiation (Scholzen and Gerdes 2000), we could not observe any difference between kidneys from *Foxc2* ^fl/fl^; Pod‐Cre^+/−^, and *Foxc2* ^fl/fl^ control mice. A slight increase in staining intensity for alpha smooth muscle actin (also known as *α*‐SMA or ACTA2*)*, a marker for mesangial cells, was observed, whereas the staining intensity of the endothelial marker CD31 was unaffected by the loss of *Foxc2* in podocytes.

### Altered distribution of proteins of the foot processes in podocyte‐specific *Foxc2* knockout mice

**Figure 5:**
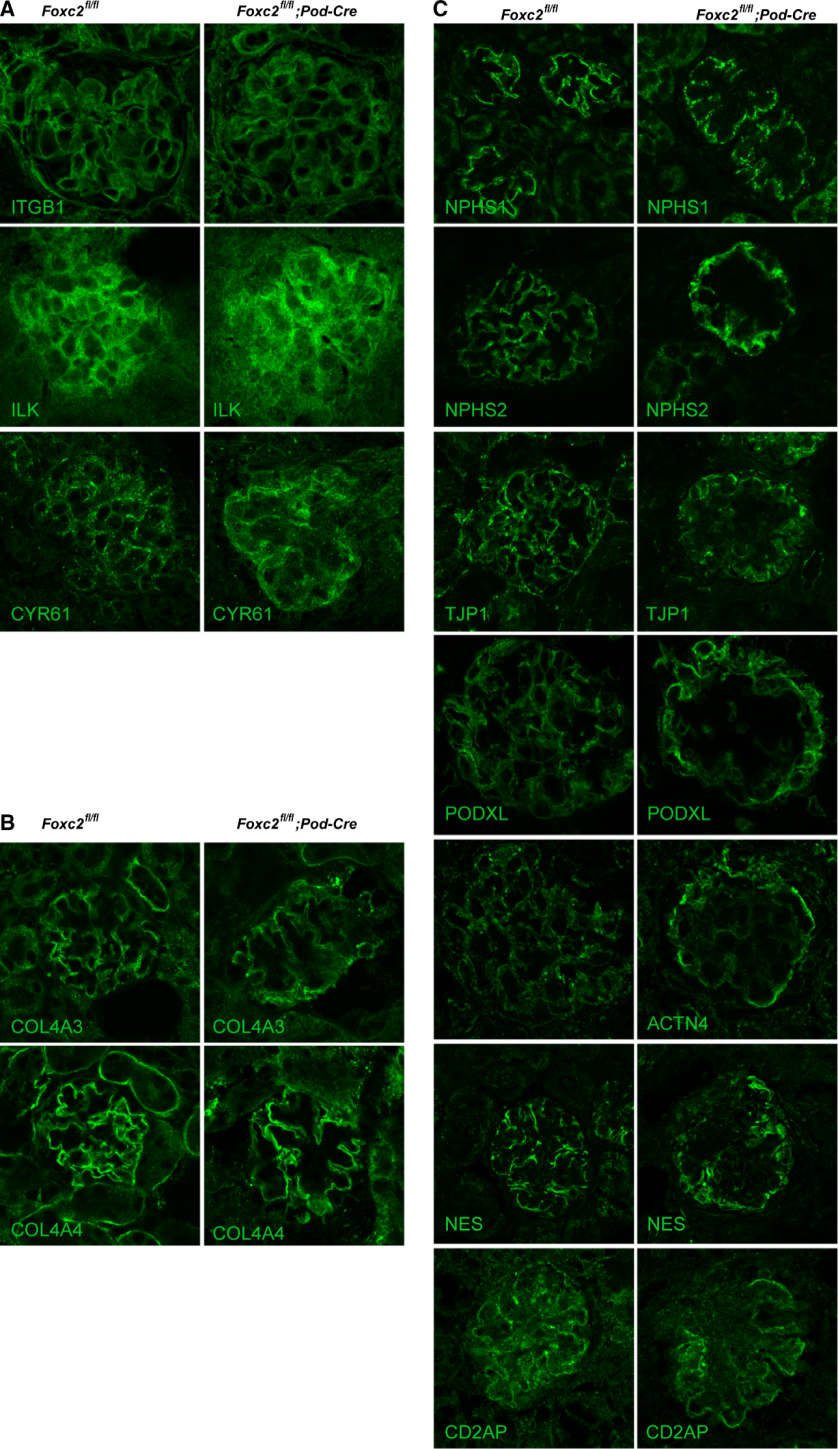
Expression of glomerular proteins. Representative laser confocal images of kidney sections from 9‐day old *Foxc2* ^*fl/fl*^;Pod‐Cre^+^ and *Foxc2* ^*fl/fl*^ mice using antibodies labeling (A) proteins involved in anchoring of podocytes (ITGB1/*β*1‐integrin, ILK/integrin linked kinase and CYR61/CCN1), (B) basement membrane proteins (COL4A3 and COL4A4), and (C) components of the foot processes (i.e., slit diaphragm proteins (NPHS1/nephrin and NPHS2/podocin), anchoring of slit diaphragm to cytoskeleton (TJP1/ZO‐1, ACTN4/*α*‐actinin 4, CD2AP/CD2‐associated protein, and NES/nestin), and foot process coating (PODXL/podocalyxin)).

To explore possible candidates for the phenotype observed in mice lacking *Foxc2* in podocytes, we examined a number of proteins that previously have been associated with podocyte damage and/or with a reported link to FOXC2. No obvious difference in expression levels could be detected for any of these (Fig. 5). Especially when labeling for proteins involved in anchoring of podocytes to the basement, like ITGB1, ILK, and CYR61 (cysteine rich angiogenic inducer 61) (Fig. 5A), but also for critical components of the basement membrane, such as COL4A3 and COL4A4 (Fig. 5B), expression appeared normal.

However, although the expression levels appear unaffected, the glomerular distribution of important components of the foot processes, such as NPHS1, NPHS2, TJP1, PODXL, ACTN4, NES (nestin), and CD2AP, was altered (Fig. 5C). Instead of lining the capillary walls of the glomerulus, as seen in healthy *Foxc2* ^fl/fl^ mice, these components are mostly limited to the outer rim of the glomeruli and are often detected in a granular pattern in podocytes of *Foxc2* ^fl/fl^; Pod‐Cre^+/−^ mice.

### Neuropilin 1 is regulated by Foxc2 in vitro

**Figure 6:**
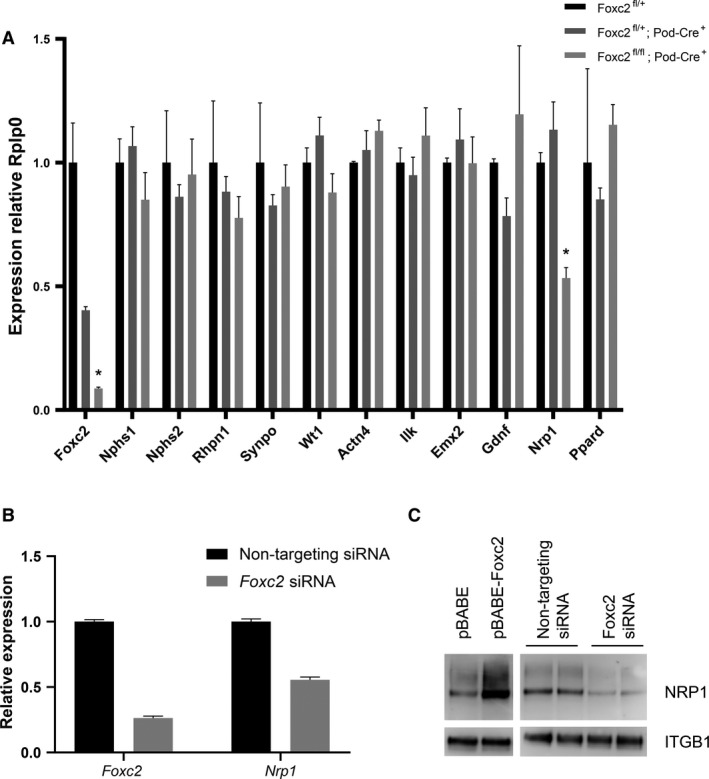
Expression of candidate genes. (A) qPCR analysis of relative mRNA levels in total RNA from isolated glomeruli of *Foxc2* ^fl/fl^; Pod‐Cre^+^, *Foxc2* ^fl/+^; Pod‐Cre^+^ and *Foxc2* ^fl/+^ mice. Expression levels were normalized against the expression of *Rplp0*. (B) qPCR analysis of relative mRNA levels in total RNA from a podocyte cell line treated with either *Foxc2* siRNA or non‐targeting siRNA. (C) Western blot analysis of NRP1 and ITGB1 protein expression after *Foxc2* induction (pBABE‐*Foxc2*) or knockdown (*Foxc2* siRNA). Empty vector (pBABE) and non‐targeting siRNA was used as controls. *P < 0.05 Foxc2^fl/fl^;Pod‐Cre^+^ versus *Foxc2* ^fl/+^. Error bars in (A) and (B) represent SEM.

To further analyze potential candidate FOXC2 targets, quantitative PCR (qPCR) was conducted. The mRNA levels in isolated glomeruli was analyzed by qPCR and, from our list of candidate genes, only neuropilin 1 (*Nrp1*) had significantly altered expression (approx. 50% lower) in glomeruli from *Foxc2* ^fl/fl^; Pod‐Cre^+/−^ mice as compared with Foxc2^fl/+^ mice (Fig. 6A).

Since glomeruli are made up of several cell types, of which podocytes only constitute about 15%, the dependence of *Foxc2* in podocytes for expression of these genes was further characterized in a podocyte cell line upon knockdown or induction of *Foxc2* expression (Schiwek et al. 2004). Transcript analysis showed that the *Foxc2* siRNA had a knockdown efficiency of about 75% on *Foxc2* mRNA. (Fig. 6B) NRP1 could be confirmed as a potential *Foxc2* target in these cells since it was co‐regulated at both the mRNA (Fig. 6B) and protein level (Fig. 6C).

**Figure 7:**
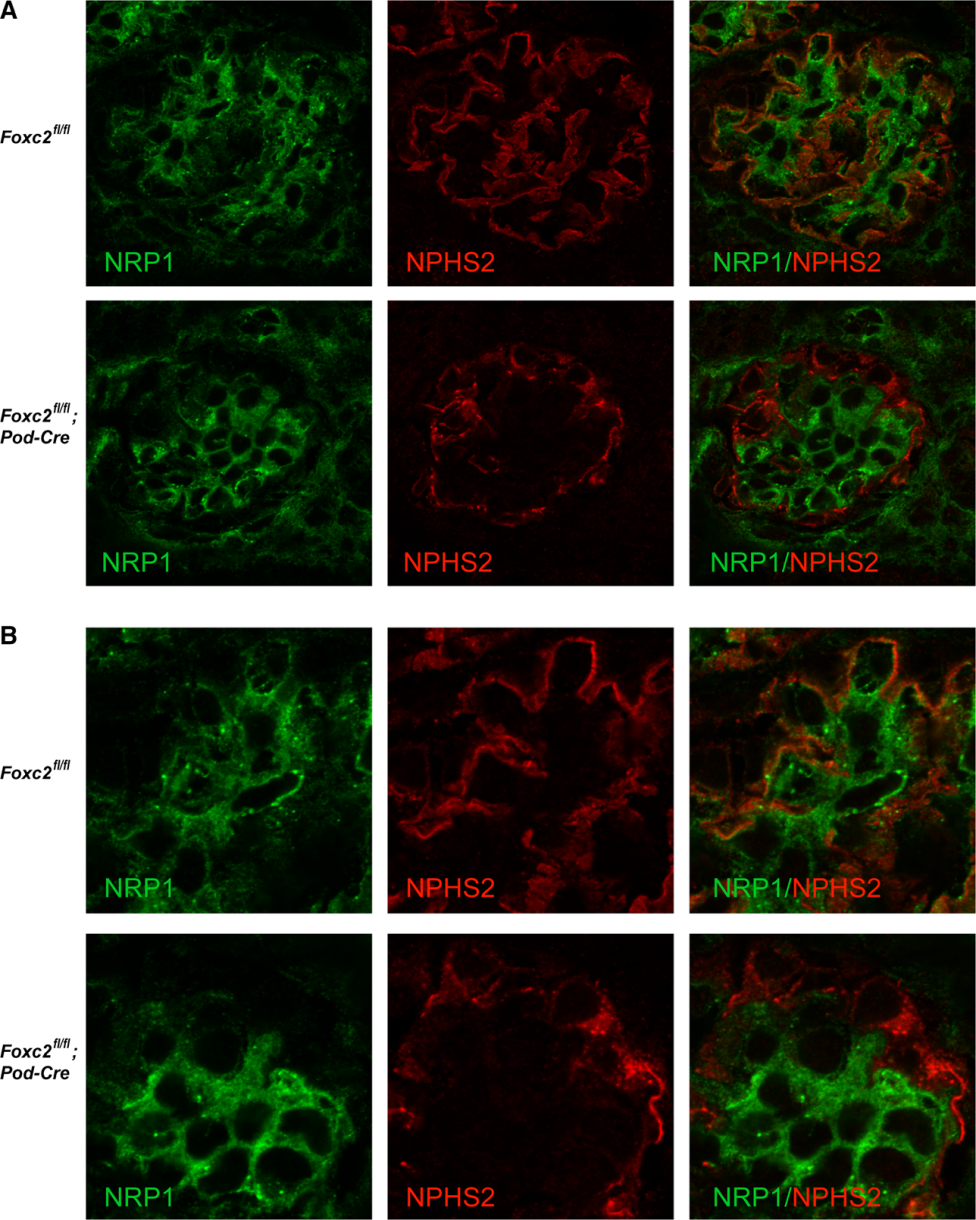
Laser confocal microscopy of kidney sections from 9‐day‐old *Foxc2* ^fl/fl^; Pod‐Cre^+^ and *Foxc2* ^fl/fl^ mice. (A) Images of whole glomeruli, with separate images of NRP1/neuropilin 1 (green) and NPHS2/podocin (red) signal and a merged image for visualization of co‐localization. Orange indicates merged/co‐localized signals. (B) Zoomed in on part of the same glomeruli as in (A) to further visualize the localization of green (NRP1) and red (NPHS2) signals.

The expression level of NRP1 in podocytes is difficult to assess in vivo, due to strong staining in adjacent endothelial cells. However, co‐staining with NPHS2, to label podocytes, reveals loss of co‐localization between NRP1 and NPHS2, and a granular distribution of NPHS2, in glomeruli of *Foxc2* ^fl/fl^; Pod‐Cre^+/−^ mice (Fig. 7) emphasizing that NPHS2 has lost its proximity to the glomerular basement membrane.

### Neuropilin 1 is not critical for podocyte function in vivo

**Figure 8:**
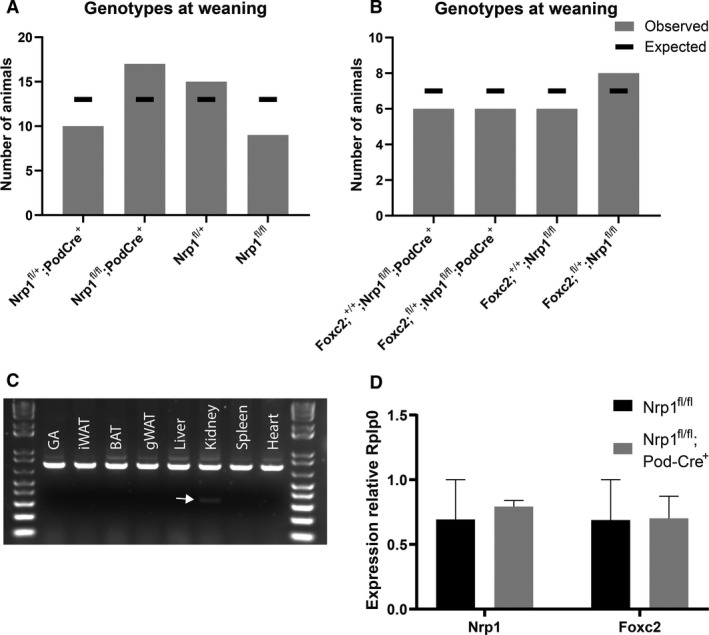
Characterization of mice with podocyte‐specific deletion of *Nrp1*. (A) Genotype ratio at weaning (3–4 weeks of age) in litters from *Nrp1* ^fl/+^; Pod‐Cre^+^ x *Nrp1* ^fl/fl^ breeding. (B) Genotype ratio at weaning (3–4 weeks of age) in litters from *Foxc2* ^fl/+^; *Nrp1* ^*fl/fl*^ *; Pod‐Cre* ^+^ × *Nrp1* ^*fl/fl*^ breeding. (C) PCR on genomic DNA using primers located outside of loxP sites. Successful Cre recombination between loxP sites enables amplification of ~350 bp fragment from *Nrp1* null allele (arrow). Amplification from wt allele generates a 739 bp fragment. Tissues analyzed: GA = gastrocnemius, iWAT = inguinal white adipose tissue, BAT = interscapular brown adipose tissue, gWAT = gonadal white adipose tissue, liver, kidney, spleen, and heart. (D) qPCR analysis of mRNA levels of *Nrp1* relative *Rplp0* in total RNA from isolated glomeruli of *Nrp1* ^fl/fl^; Pod‐Cre^+^ and *Nrp1* ^fl/fl^ control mice. Error bars represent SEM.

To evaluate the role of NRP1 in podocytes and its potential contribution to the observed phenotype of *Foxc2* ^fl/fl^; Pod‐Cre^+/−^ animals, mice with floxed *Nrp1* (Gu et al. 2003), were crossed with Pod‐Cre^+/−^ transgenic mice to create podocyte‐specific knockout of *Nrp1*. All *Nrp1* ^fl/fl^; Pod‐Cre^+/−^ mice were viable and appeared healthy (Fig. 8A). Not even when provoking the genotype with the addition of a floxed *Foxc2* allele, that is, adding a heterozygous deletion of *Foxc2* to the homozygous deletion of *Nrp1* in podocytes, there were signs of premature death (Fig. 8B). To confirm deletion of floxed *Nrp1* in podocytes, genomic DNA from various tissues from *Nrp1* ^fl/+^; Pod‐Cre^+/−^ mice was analyzed by PCR. Only in gDNA from kidney of these mice ~350 bp PCR product could be amplified, using primers located outside of the floxed sequence (Fig. 8C) and given the podocyte‐specific expression of Cre recombinase it most likely corresponds to deletion in podocytes. Surprisingly, podocyte‐specific knockout of *Nrp1* did not reduce glomerular mRNA levels of *Nrp1* (Fig. 8D).

Although the amount of *Nrp1* in glomeruli was not reduced, the levels in podocytes should be eradicated in this model. To examine whether this causes podocyte injury, urine samples were collected from both young (1 month old) and older mice (14 months old) and analyzed for microalbuminuria, an indicator of podocyte injury, using ELISA. Still, there was no difference in albumin content in the urine of the different genotypes no matter what age or gender analyzed (data not shown), suggesting a minor (if any) contribution of NRP1 to podocyte function.

## Discussion

Podocytes have been shown to express the forkhead transcription factor *Foxc2* (Dagenais et al. 2004; Takemoto et al. 2006; Brunskill et al. 2011), which appears as one of the earliest markers of podocytes during glomerular development (Takemoto et al. 2006). Characterization of mice with global *Foxc2* knockout revealed several kidney defects, including reduced kidney size, abnormal glomerular shape, dilated and blood‐filled capillary loops, and failure to produce a proper mesangial core of the glomeruli (Takemoto et al. 2006). In addition, podocytes lacked foot processes and slit diaphragms, and endothelial cells lacked fenestrations. In this model, all mice die in utero or around birth, hence precluding functional analysis of the kidney.

Using in situ hybridization and immunohistochemistry, Takemoto et al. (2006) showed that, in embryonic kidney, FOXC2 was mainly expressed in podocytes, suggesting that the histological defects seen in the kidney were derived from arrested podocyte development. However, we show here, using the *Foxc2* ^lacZ/+^ mouse model (Cederberg et al. 2009), that *Foxc2* expression in the kidney is not exclusive to podocytes, but is evident also in other structures. The use of a *lacZ* – marker for endogenous *Foxc2* expression is likely to provide a more sensitive detection method than immunohistochemistry and in situ hybridizations. The additional embryonic expression of *lacZ* also correlates well with previously reported expression pattern of *Foxc2* (Miura et al. 1993; Kaestner et al. 1996; Takemoto et al. 2006), which further validates the *Foxc2* ^lacZ/+^ model.

To study the specific role of FOXC2 in the podocyte, we generated a floxed *Foxc2* mouse that was crossed with Pod‐Cre transgenic mice, a commonly used Cre‐transgenic strain for podocyte‐specific deletions (Moeller et al. 2003; El‐Aouni et al. 2006; Suleiman et al. 2007; Pozzi et al. 2008). In contrast to the global deletion of *Foxc2*, the conditional deletion of *Foxc2* in podocytes did not cause embryonic lethality, but the animals developed heavy proteinuria soon after birth (detected already in 5‐day‐old pups), and died around postnatal day 10 due to severe kidney failure. Importantly, there were no indications of kidney dysfunction in terms of albuminuria until postnatal day 5. This finding is in bright contrast to a recent work by Motojima et al. 2017 where *Foxc2* was conditionally deleted in podocytes, using Nephrin‐Cre. In this model, no kidney phenotype was observed unless challenged with combinatorial deletion of Foxc1. It is surprising that the phenotypes are so different. Especially, since nephrin expression appears earlier than podocin during podocyte differentiation (Putaala et al. 2001; Takemoto et al. 2006) with increased risk of developmental effects. Given that Foxc2 is a single‐exon gene, both models are required to target the same exon. However, there appears to be a slight difference in the location of the loxP sites in the two models. Motojima et al. inserted the 3′ loxP site within the 3′ UTR (untranslated region) of Foxc2 (Motojima et al. 2016b), whereas we intentionally avoided this transcribed region due to the risk of affecting crucial, but yet unknown, regulatory elements. Another, perhaps more plausible explanation, could be that the penetrance of Cre expression in podocytes is different in the two transgenic strains utilized by the different groups. We could show substantial reduction in *Foxc2* mRNA in glomeruli of *Foxc2* ^fl/fl^; Pod‐Cre^+/−^ mice; but unfortunately no such data were presented by Motojima et al. Hence, it is difficult to compare data from these two models since there might be differences in knockout efficiency.

Ultrastructural analysis of kidneys from *Foxc2* ^fl/fl^; Pod‐Cre^+/−^ mice confirmed that podocytes lacking *Foxc2* develop foot process effacement, a common, but nonspecific feature of podocyte injury, as well as extensive podocyte microvillus transformation, another manifestation of podocyte damage (Patek et al. 1999; Roselli et al. 2004; El‐Aouni et al. 2006). Interestingly, the podocytes in *Foxc2* ^fl/fl^; Pod‐Cre^+/−^ mice seem to develop properly since at postnatal day 3, the presence of interdigitated foot processes and normal urinary albumin concentrations could be demonstrated. Other typical signs of glomerular injury, such as thickening of the basement membrane or loss of fenestrated endothelial cells, the latter a feature of the global *Foxc2* knockout mouse (Takemoto et al. 2006), could not be detected in *Foxc2* ^fl/fl^; Pod‐Cre^+/−^ mice; not even at day 9 when the mice suffer from end‐stage kidney failure. Additionally, in contrast to findings in the global *Foxc2* knockout mice, podocyte‐specific *Foxc2* deletion did not affect kidney size.

Loss of *Foxc2* in podocytes does not affect podocyte number, proliferation/dedifferentiation or apoptosis, conditions known to cause foot process effacement and proteinuria (Greka and Mundel 2012). There are signs of mesangial expansion at the late stage of renal failure in these mice and this might be an indication of progressive glomerular injury (Dalla Vestra et al. 2001). Notably, the mesangial expansion seen in the podocyte‐specific *Foxc2* knockout mice is the opposite effect of what was observed in the global knockout of *Foxc2*, where the mesangial core failed to develop properly (Takemoto et al. 2006). This inequality adds to the conclusion that these two models have distinct phenotypes, one a general developmental phenotype and one a specific podocyte function phenotype.

To investigate potential *Foxc2* targets, we pursued a candidate gene approach based on either phenotypic similarities and/or a reported link to *Foxc2*. The importance for proper glomerular function have been reported for NPHS2 (Boute et al. 2000; Roselli et al. 2004), TJP1 (Itoh et al. 2014), PODXL (Doyonnas et al. 2001), COL4A3 (Miner and Sanes 1996), COL4A4 (Korstanje et al. 2014), and RHPN1 (Lal et al. 2015), targets that were all downregulated in glomeruli of the global *Foxc2* knockout mouse (Takemoto et al. 2006). In addition to these genes, we analyzed targets that share phenotypic similarities with *Foxc2* and have an established function or expression in podocytes, that is, NPHS1 (Lenkkeri et al. 1999), ACTN4 (Kos et al. 2003), NES (Perry et al. 2007), and CD2AP (Shih et al. 2001).

Unexpectedly, none of these proteins showed detectable changes in expression levels in podocyte‐specific *Foxc2* knockout mice when examined by immunohistochemistry. This was particularly surprising for those candidates that were affected in the global knockout of *Foxc2*. However, this finding shows many similarities with studies of another transcription factor, LMX1B, where proposed downstream targets derived from a global knockout model, that is, NPHS2, COL4A3, and COL4A4, could not be confirmed when conditionally deleting *Lmx1b* in podocytes (Suleiman et al. 2007; Burghardt et al. 2013). This further emphasizes the benefit of studying the in vivo role of genes in cell‐specific knockouts rather than global deletions, since effects on the cellular level observed in global knockouts might be derived from accumulated or developmental effects.

One striking feature observed after conditional deletion of *Foxc2* in podocytes is the change in distribution of components of the foot process. Such components normally accumulate along the capillary lining, being associated with the basement membrane, but was observed in a cytoplasmic, often granular, and rim‐like pattern in the *Foxc2*‐depleted podocytes. A change in distribution of podocyte markers is often observed after foot process effacement (Shirato 2002). Proteins that are involved in the anchoring of the podocyte (ITGB1 and ILK) or constituting components of the basement membrane (COL4A3 and COL4A4) do not share this altered expression pattern. A thorough investigation of other anchoring or basal lamina components, like integrins and laminins, was performed on *Foxc2* global knockout mice, without any detectable changes (Takemoto et al. 2006). These findings, together with the ultrastructural analyses, suggest that *Foxc2* expression in podocytes is crucial to maintain the architecture of foot processes.

We identified *Nrp1*, a transmembrane co‐receptor that can bind either VEGF or semaphorins (Gu et al. 2003), as a downstream *Foxc2* target in vitro. A link between these two genes has already been proposed in endothelial cells (Hayashi and Kume 2008), so we investigated the glomerular expression of NRP1. Strikingly, the co‐localization that NRP1 possess with the podocyte‐specific NPHS2 in healthy kidneys was completely lost in podocytes that lacked *Foxc2*. Encouraged by these findings, and the many indications that NRP1 might have an important role in podocytes and the development of proteinuria (Bondeva et al. 2009; Loeffler et al. 2013; Patnaik et al. 2014; Bondeva and Wolf 2015), we pursued this target further. Surprisingly, conditional deletion of *Nrp1* in podocytes in vivo did not cause any kidney‐related effects at all. It did not even reduce glomerular mRNA levels of *Nrp1*, which suggests that the reduced levels that were observed in the glomeruli from *Foxc2* conditional knockouts might be derived from glomerular cells other than podocytes. Increased *Nrp1* expression has also been shown to lead to increased leakage of vessels (Roth et al. 2016), which might even indicate that reduced levels of *Nrp1* in endothelial cells is a response to decrease the leakage in the glomeruli. A recent report also shows that expression of *Nrp1* in mesangial cells is vital for proper glomerular function when deleted using *Pdgfrb*‐*Cre* (Bartlett et al. 2017), indicating that *Nrp1* are more likely to play a role in mesangial cells rather than podocytes.

To conclude, we show for the first time that *Foxc2*, but not *Nrp1*, expression is essential for proper podocyte function in vivo. We could clearly demonstrate that podocytes that are deprived from *Foxc2* lose their unique architecture and hence their ability to prevent proteins from leaking into the urine. Nevertheless, despite the dramatic phenotype, it is not associated with detectable alterations in levels of important glomerular components, and previously proposed targets, including NPHS2, COL4A3, COL4A4, or *Rhpn1*. Further studies will therefore be needed to identify genes regulated by *Foxc2* in podocytes and how *Foxc2* regulates them during normal kidney physiology to maintain proper podocyte structure and function.

## Conflict of Interest

None.

## Supporting information




**Figure S1.** Strategy for generation of mice with conditional‐ready floxed *Foxc2* allele.
**Figure S2.** Analysis of urinary albumin concentration in young and old mice.
