# Supplementary material for: Foxc2 is essential for podocyte function
Source: Physiol Rep. 2019 May 6;7(9):e14083. doi: 10.14814/phy2.14083 (PMC6503019; doi:10.14814/phy2.14083)

SUPPORTING INFORMATION

SUPPORTING FIGURE LEGENDS

Supporting Figure 1. Strategy for generation of mice with conditional-ready floxed *Foxc2* allele.

See METHODS for detailed description. neo; neomycin cassette, DTA; difteria toxin A cassette, black triangle; loxP sequence, black bar; probe for Southern blot, half arrow; primer for genotyping, BamHI/NcoI/XbaI/SacI/HindIII; restriction enzyme sites used for cloning or Southern blot.

Supporting Figure 2. Analysis of urinary albumin concentration in young and old mice.

Urine from mice with podocyte-specific *Nrp1* deletion and littermate controls were analyzed. Albumin concentration in urine from (A) young mice (1 month old, mixed gender), (B) old male mice (14 months old) or (C) old female mice (14 months old) was measured by ELISA. Individual albumin concentrations (µg albumin/ml urine) are grouped based on genotype.

Line indicates mean value of the group. Data from aged mice were separated by gender in this figure due to a more progressed albuminuria in male mice.

Supporting Figure 1

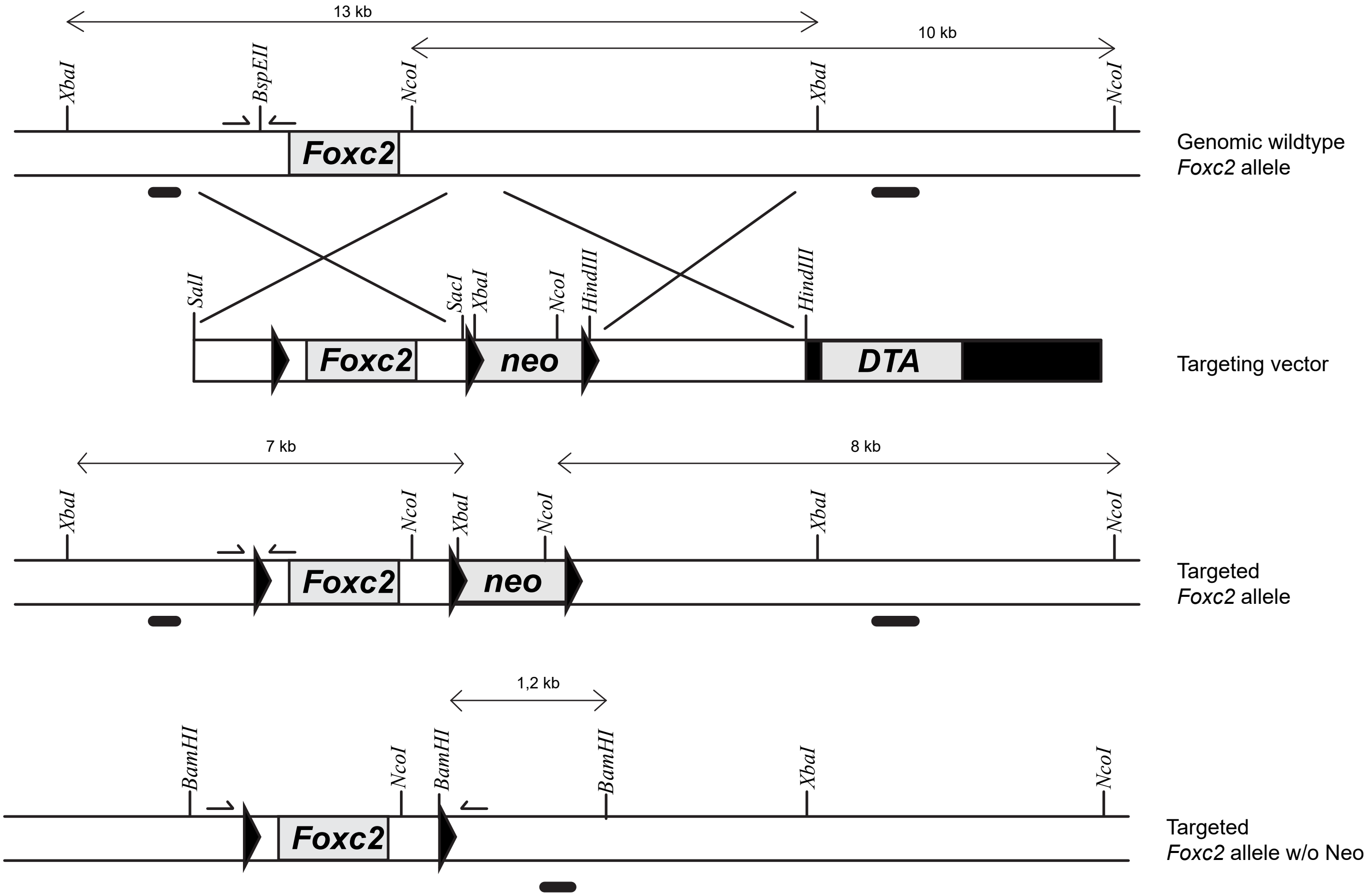

Supporting Figure 2

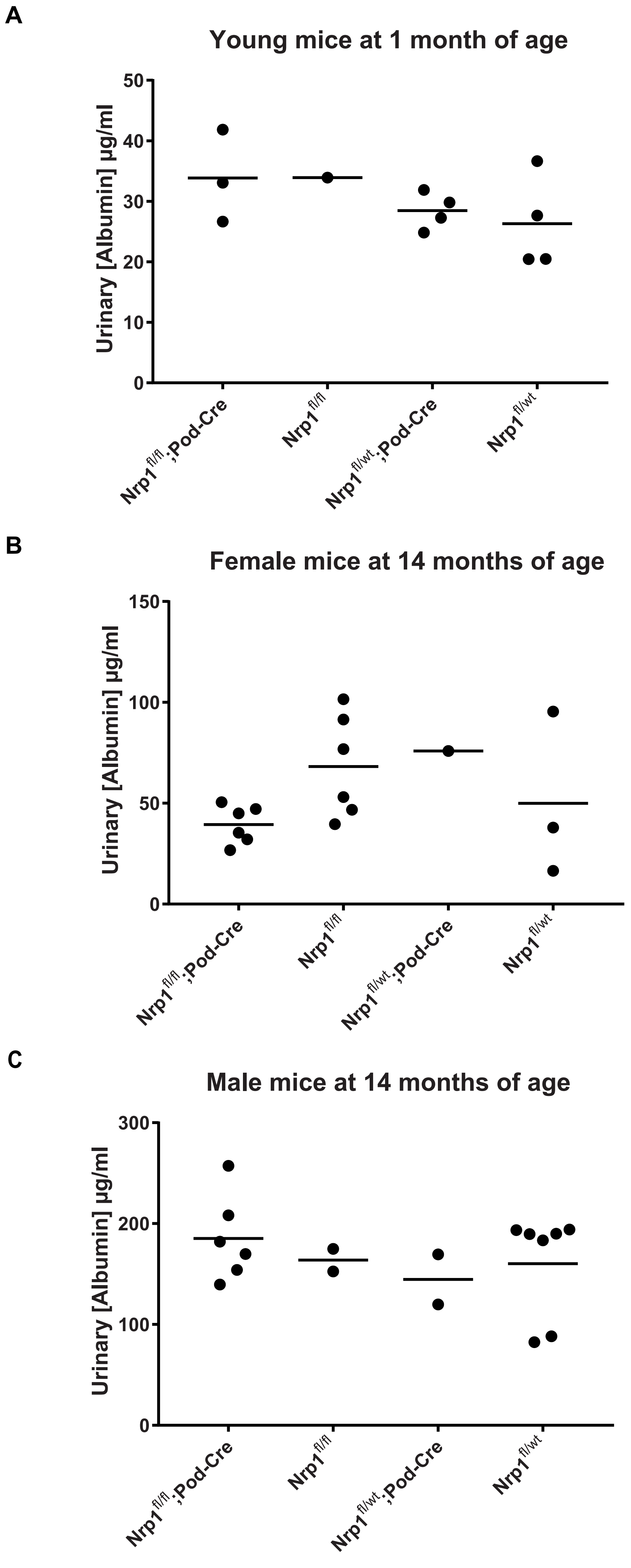

Supplement: Supplementary file 1 — Figure S1. Strategy for generation of mice with conditional‐ready floxed Foxc2 allele. Figure S2. Analysis of urinary albumin concentration in young and old mice. [file PHY2-7-e14083-s001.pdf]
